# Engineering Surface Oxophilicity of Copper for Electrochemical CO_2_ Reduction to Ethanol

**DOI:** 10.1002/advs.202204579

**Published:** 2022-11-17

**Authors:** Minhan Li, Nan Song, Wei Luo, Jun Chen, Wan Jiang, Jianping Yang

**Affiliations:** ^1^ State Key Laboratory for Modification of Chemical Fibers and Polymer Materials College of Materials Science and Engineering Donghua University Shanghai 201620 P. R. China; ^2^ College of Materials Science and Engineering Zhengzhou University Zhengzhou 450001 P. R. China; ^3^ State Key Laboratory of Chemical Engineering East China University of Science and Technology Shanghai 200237 P. R. China; ^4^ ARC Centre of Excellence for Electromaterials Science Intelligent Polymer Research Institute Australian Institute of Innovative Materials University of Wollongong Innovation Campus Wollongong NSW 2522 Australia

**Keywords:** C_2_H_5_OH pathway, CO_2_RR, Cu‐Sn bimetallic catalyst, reaction mechanism, surface oxophilicity

## Abstract

Copper‐based materials are known for converting CO_2_ into deep reduction products via electrochemical reduction reaction (CO_2_RR). As the major multicarbon products (C_2+_), ethanol (C_2_H_5_OH) and ethylene (C_2_H_4_) are believed to share a common oxygenic intermediate according to theoretical studies, while the key factors that bifurcate C_2_H_5_OH and C_2_H_4_ pathways on Cu‐based catalysts are not fully understood. Here, a surface oxophilicity regulation strategy to enhance C_2_H_5_OH production in CO_2_RR is proposed, demonstrated by a Cu‐Sn bimetallic system. Compared with bare Cu catalyst, the Cu‐Sn bimetallic catalysts show improved C_2_H_5_OH but suppressed C_2_H_4_ selectivity. The experimental results and theoretical calculations demonstrate that the surface oxophilicity of Cu‐Sn catalysts plays an important role in steering the protonation of the key oxygenic intermediate and guides the reaction pathways to C_2_H_5_OH. This study provides new insights into the electrocatalyst design for enhanced production of oxygenic products from CO_2_RR by engineering the surface oxophilicity of copper‐based catalysts.

## Graphical abstract

Engineering the surface oxophilicity of copper‐based catalysts is a promising way to increase the selectivity of ethanol in electrochemical CO_2_ reduction reaction. The introduction of oxophilic Sn metal enhances the surface oxophilicity of Cu‐Sn bimetallic catalyst, which improves the ethanol production compared to bare Cu catalyst.

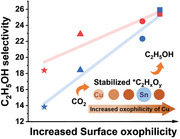

## 1 Introduction

The electrochemical CO_2_ reduction reaction has been attracting worldwide attention due to its great potential to convert CO_2_ and H_2_O into valuable chemicals using renewable power.^[^ ^1^ ^]^ CO_2_ is a stable and inert molecule, which makes it essential to develop highly efficient catalysts to lower the energy barrier of CO_2_RR process.^[^ ^2^ ^]^ Cu‐based materials stands out as a unique kind of catalysts because of their selectivity toward multicarbon (C_2+_) products.^[^ ^3^ ^]^ Extensive efforts have been made to increase the Faradaic efficiency (FE) of total C_2+_ products.^[^ ^4^ ^]^ As the major C_2+_ products, C_2_H_4_ and C_2_H_5_OH are both 12‐electron transfer products while C_2_H_5_OH usually exhibits lower selectivity than C_2_H_4_ on Cu‐based catalysts in neutral electrolyte of CO_2_RR.^[^ ^5^ ^]^ Ethanol is an important liquid fuel with high energy density (26.8 MJ kg^−1^). To enhance the production of C_2_H_5_OH from CO_2_RR, several strategies have been proposed, such as tandem effect,^[^ ^6^ ^]^ electronic effect,^[^ ^7^ ^]^ strain effect,^[^ ^8^ ^]^ adsorbed hydrogen assistance,^[^ ^9^ ^]^ and binding site diversification.^[^ ^5a^ ^]^

The surface oxophilicity has been found to play a key role in tuning the binding strength of the oxygenic intermediates and thereby affecting the activity and selectivity of many O‐bond relevant electrocatalytic processes, such as hydrogen evolution reaction (HER), oxygen evolution reaction (OER), oxygen reduction reaction (ORR), and ethanol oxidation reaction (EOR).^[^ ^10^ ^]^ For CO_2_RR catalyzed by Cu‐based catalysts, multiple oxygenic intermediates are involved on the reaction pathways to various deep reduction products. The oxophilicity of Cu‐based catalysts has been proposed to play a key role in promoting carbonyl‐containing products or branching the reaction pathway toward methane and methanol.^[^ ^11^ ^]^ As for C_2_ products, although the pathways for the formation of C_2_H_4_ and C_2_H_5_OH have not reached a consensus, both theoretical and experimental researches have suggested that the formation of C_2_H_4_ and C_2_H_5_OH branch after CO dimerization and they share a key oxygenic species.^[^ ^7^, ^12^ ^]^ This oxygenic intermediates can be referred as the selectivity‐determining intermediate (SDI), whose next proton and electron transfer bifurcates the pathways for C_2_H_4_ and C_2_H_5_OH.^[^ ^7^, ^12^, ^13^ ^]^ To stabilize *CH_2_CHO intermediate and promote C_2_H_5_OH pathway, a catalyst contains dual active sites was recently proposed to enhance the production of C_2_H_5_OH by introducing strong oxophilic nitrogen‐doped graphene.^[^ ^14^ ^]^ Therefore, such adsorbate binding characteristics regulated by the surface oxophilicity of Cu‐based catalysts holds the promise to enhance C_2_H_5_OH production in CO_2_RR. However, the way to efficiently regulate the surface oxophilicity of Cu‐based catalysts is still lacking and how the surface oxophilicity affect the selectivity of C_2_ products in CO_2_RR remains unclear.

Bimetallic strategy that combines two elements with different oxophilicity is a promising way to regulate the oxophilicity of metal‐based catalysts.^[^ ^15^ ^]^ According to the oxophilicity of the metals reflected by the bond dissociation enthalpies of metal–oxygen (Figure S1, Supporting Information),^[^ ^16^ ^]^ Sn is a wise choice to couple with Cu because of the greater oxophilicity of Sn and the formate selectivity of Sn in CO_2_RR.^[^ ^17^ ^]^ The Cu‐Sn bimetallic catalysts have been extensively investigated in CO_2_RR and exhibited high activity and selectivity of two‐electron (2e) products (Table S1, Supporting Information) at low and moderate overpotentials (from −0.5 to −1.1 V_RHE_ in literatures).^[^ ^18^ ^]^ Although some studies have suggested small amount of deep reduction products were generated on Cu‐Sn bimetallic catalysts at moderate overpotentials,^[^ ^19^ ^]^ the CO_2_RR performance of Cu‐Sn bimetallic catalysts at large overpotentials remains unexplored.

In this study, we focus on the influence of surface oxophilicity of Cu‐based catalysts on CO_2_RR performance. To regulate the surface oxophilicity of Cu, a series of dendrite Cu‐Sn bimetallic catalysts is synthesized by a facile replacement method, featuring surface‐enriched Sn incorporated in Cu lattice. Compared to Cu, the Cu‐Sn bimetallic catalysts exhibited enhanced C_2_H_5_OH selectivity but suppressed C_2_H_4_ selectivity at relatively large overpotential. The effects of Sn modification on the oxophilicity and C_2_ products pathways on CuSn*_x_* catalysts were studied by both experiments and theoretical calculations. An insightful understanding of the effect of surface oxophilicity on C_2_H_5_OH selectivity in CO_2_RR was proposed, suggesting the surface oxophilicity regulation of Cu‐based catalysts is a promising way to promote C_2_H_5_OH production in CO_2_RR.

## 2 Results and Discussion

### 2.1 Synthesis and Characterization of CuSn*_x_*

**Figure 1:**
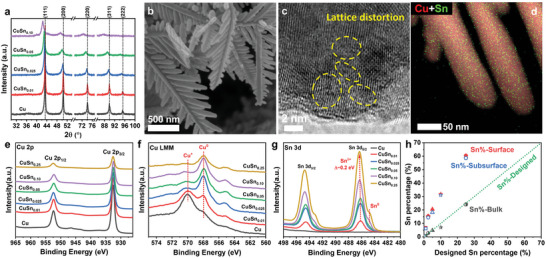
Materials characterizations. a) XRD patterns of CuSn*_x_* catalysts. b) SEM image of CuSn_0.01_ catalyst. c) HRTEM images of CuSn_0.01_ catalyst. d) The overlapped HAADF‐STEM images and EDS elemental mappings of CuSn_0.01_ catalyst. e) Cu 2p XPS spectra of CuSn*_x_* catalysts. f) Cu LMM Auger spectra of CuSn*_x_* catalysts. g) Sn 3d XPS spectra of CuSn*_x_* catalysts. h) Bulk and surface composition characterized by EDS and XPS.

The Cu‐Sn bimetallic catalysts were synthesized by a rapid and facile method that based on simultaneous galvanic replacement reactions (Figure S2a, Supporting Information). Driven by the difference in the redox potential, this galvanic replacement process is a powerful method for fabricating dendritic structures with controllable chemical composition.^[^ ^20^ ^]^ Once a piece of cleaned Zn foil was added into a mixed solution containing Cu^2+^, Sn^2+^, and HCl, the surface of silver gray Zn foil turned into black immediately, indicating the reaction took place rapidly (Figure S2b, Supporting Information). The Zn foil almost disappeared and the color of the solution became light after 1 h, indicating the deposition of Cu and Sn as well as the dissolution of Zn^2+^. The obtained solid products were immersed in diluted HCl solution and then washed with deionized water repeatedly to remove the superfluous Zn^2+^. The X‐ray diffraction (XRD) patterns of CuSn*_x_* catalysts with *x* ≤ 0.10 showed similar diffraction peaks with bare Cu catalyst while no crystalline Sn species were detected (Figure S3a, Supporting Information). As seen in **Figure** **1**a, with the increasing content of Sn, all the diffraction peaks shifted to lower 2*θ* angle and the peaks became widened, indicating the enlarged lattice spacing and decreased crystallinity and grain size.^[^ ^18a^ ^]^ These results are likely due to the Sn atoms with larger atomic radius than Cu atoms are highly dispersed in the lattice of Cu, which will be further discussed below.^[^ ^21^ ^]^ Furthermore, the XRD pattern of CuSn_0.25_ catalyst showed that the intermetallic phase corresponding to Cu_41_Sn_11_ emerged when the Sn content further increased to 25% (Figure S3b, Supporting Information).

The morphologies of the CuSn*_x_* bimetallic catalysts were first characterized by scanning electron microscope (SEM). For comparison, bare Cu sample was also prepared by the same method without Sn^2+^ addition. The bare Cu counterpart exhibited irregular rod‐like morphology (Figure S4a, Supporting Information). With the introduction of Sn, the microstructure of CuSn*_x_* catalysts exhibited typical dendritic morphology (Figure 1b and Figure S4b–f, Supporting Information), indicating that Sn^2+^ ions may facilitate the formation of dendritic morphology in the replacement process. The dendrite structure is believed to be beneficial to CO_2_RR process by lowering the charge transfer resistance and enhancing the mass transfer.^[^ ^19^, ^22^ ^]^ The double‐layer capacities of the CuSn*_x_* catalysts were then measured to determine the electrochemical surface area (ECSA). As expected, CuSn*_x_* catalysts exhibited similar ECSA except for CuSn_0.25_, which is higher bare Cu catalyst (Figure S5, Supporting Information). Representative high‐resolution transmission electron microscope (HRTEM) images showed dendritic structure and good crystallinity of CuSn_0.01_ catalyst (Figure S6, Supporting Information). The zoomed‐in edge of CuSn_0.01_ catalyst exhibited distorted lattice fringe, which is likely due to the heteroatomic doping of Sn in Cu lattice (Figure 1c). The elemental mapping of CuSn_0.01_ catalyst showed the predominant Cu and highly scattered Sn elements were uniformly distributed in the catalyst (Figure 1d and Figure S7, Supporting Information). On the other hand, the TEM images of CuSn_0.10_ catalyst showed that the coarsened dendrite surface become assembly of small particles and a distinct amorphous layer of about 5 nm formed on the catalyst surface (Figure S8a–c, Supporting Information). The elemental mapping of CuSn_0.10_ catalyst showed a uniform distribution of both Cu and Sn elements (Figure S8d–g, Supporting Information).

The X‐ray photoelectron spectroscopy (XPS) measurement was then carried out to investigate the surface properties of CuSn*_x_* catalysts. The Cu 2p_1/2_ and Cu 2p_3/2_ peaks of the CuSn*_x_* catalysts located at 952.4 and 932.5 eV, respectively, which can be assigned to either Cu^0^ or Cu^1+^ (Figure 1e). The Cu LMM Auger electron spectra suggested that Cu^1+^ was the main Cu species in bare Cu catalysts due to the spontaneous oxidation under ambient condition. However, with the increasing Sn content in CuSn*_x_*, the valance of Cu gradually shifted to metallic Cu^0^, which is likely due to the stronger O affinity of Sn than Cu (Figure 1f). The doublet peaks of Sn 3d (Figure 1g) that can be assigned to the 3d_3/2_ and 3d_5/2_ peaks centered at 494.6 and 486.3 eV was in between the peaks of Sn^2+^ and Sn^4+^, indicating the oxidation state of Sn^*δ*+^ in the CuSn*_x_* catalysts. The peak shift for both Cu and Sn demonstrated the electronic interaction between incorporated Sn and Cu matrix in the CuSn*_x_* catalysts. Furthermore, a small peak at about 485.0 eV that assigned to metallic Sn^0^ in Sn 3d_5/2_ peak (493.2 eV for Sn 3d_3/2_ peak) appeared in CuSn_0.10_ and CuSn_0.25_ catalysts. The composition of the CuSn*_x_* catalysts was characterized by both energy dispersive spectroscopy (EDS) and XPS. Given the nanoscale of the CuSn*_x_* catalysts, the Cu/Sn ratios determined by EDS reflect the bulk composition of the catalysts, which were close to the designed values (Figure 1h). However, the surface and subsurface Cu/Sn ratio in depth of a few nanometers determined by XPS measurements were quite different from the EDS results. The Cu/Sn ratio in the CuSn*_x_* catalysts decreases in the order: surface (XPS) > subsurface (XPS after Ar sputtering) ≫ bulk (EDS). Furthermore, the weight percentage of Cu and Sn in CuSn*_x_* catalysts was also probed by inductively coupled plasma atomic emission spectrometry (ICP‐AES), which showed Cu/Sn ratios close to the bulk (Table S1, Supporting Information). These results indicate that the Sn species enriched in the surface of the dendrite CuSn*_x_* catalysts, which is crucial for the catalytic reaction.^[^ ^19a^ ^]^

### 2.2 CO_2_RR Performance

**Figure 2:**
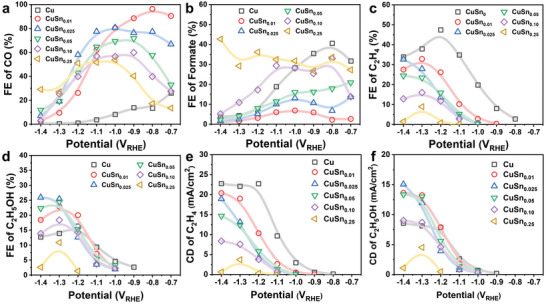
CO_2_RR performance of CuSn*_x_*. a) FE of CO. b) FE of formate. c) FE of C_2_H_4_. d) FE of C_2_H_5_OH. e) Partial current density of C_2_H_4_. f) Partial current density of C_2_H_5_OH.

The CO_2_RR performance of the dendrite CuSn*_x_* catalysts was evaluated in a H‐cell divided by a Nafion membrane. The calculated FEs of all products on different CuSn*_x_* catalysts were in the range of 93%–101% (Figure S9, Supporting Information). The FEs of different reduction products are plotted against potentials to show the change of product distributions with CuSn*_x_* catalysts (**Figure** **2** and Figure S10, Supporting Information). The competing HER was largely suppressed for all the CuSn*_x_* catalysts at the tested potentials apart from only a few exceptions at −0.7 V_RHE_ (Figure S10a, Supporting Information). As reported by many previous studies, the introduction of Sn greatly improved the selectivity of Cu‐based catalysts and CO became the major reduction product at low and moderate overpotentials.^[^ ^18d^ ^]^ Among all the CuSn*_x_* catalysts, CuSn_0.01_ exhibited the highest FE of 96.4% for CO with a partial current density of 6.50 mA cm^−2^ at −0.8 V_RHE_ (Figure 2a and Figure S11b, Supporting Information). The FEs of CO decreased gradually with the increasing Sn content and the maximum FEs of CO for CuSn_0.025_, CuSn_0.05_, CuSn_0.10_, and CuSn_0.25_ were 80.6%, 71.7%, 59.8%, and 53.7%, respectively. As another 2e transfer products, the FEs of formate increased with the increasing Sn content in CuSn*_x_* catalysts, which is due to the surface‐enriched Sn and the high formate‐producing activity of Sn in CO_2_RR (Figure 2b). As mentioned before, the suppressed HER and enhanced 2e products (CO and formate) have been extensively observed and discussed by many studies on bimetallic Cu‐Sn catalysts. Sn metal possess a weaker H affinity than Cu metal and SnO_2_ modification was reported to lower the H_2_ chemisorption of CuO.^[^ ^18^, ^23^ ^]^ Therefore, it is commonly accepted that the surface decorated Sn atoms on Cu disfavor the adsorption of *H and thereby suppress the HER.^[^ ^22^, ^24^ ^]^ Meanwhile, the formation of the important *COOH intermediate is facilitated, while the adsorption of CO is either weakened or unaffected by the combination of Cu and Sn.^[^ ^18^, ^24^ ^]^ Therefore, the enhanced production of 2e products is usually reported in previous studies about Cu‐Sn bimetallic catalysts. Similarly, a recent study showed that Cu_99_Sn_1_ catalyst with only 1% of Sn exhibited a high CO selectivity over 90% while Cu_70_Sn_30_ catalyst exhibited a high formate selectivity at low and moderate overpotentials.^[^ ^19a^ ^]^

We then continued the CO_2_RR tests at more negative potentials where deep reduction products are generated. The bare Cu catalyst exhibited the highest FEs and current densities of C_2_H_4_ in the whole potential range (Figure 2c,e). Clearly, the formation of C_2_H_4_ on Cu catalysts was suppressed by Sn modification. With the increasing Sn content in CuSn*_x_* catalysts, not only the FEs of C_2_H_4_ decreased, but also the onset potentials for C_2_H_4_ formation lowered. The onset potentials of C_2_H_5_OH on the CuSn*_x_* catalysts were also lower than that on the bare Cu catalyst. However, the FEs of C_2_H_5_OH on CuSn_0.01_, CuSn_0.025_, and CuSn_0.05_ catalysts were higher than that on Cu catalyst at potentials below −1.2 V_RHE_ (Figure 2d and Figure S12, Supporting Information). The highest FE of C_2_H_5_OH reached 25.93% on CuSn_0.025_ at −1.4 V_RHE_ with a large partial current density of 15.05 mA cm^−2^ (Figure 2d,f, Supporting Information). It is worth noting that, at such high overpotentials, the current density was still stable and the surface composition and dendrite structure of CuSn_0.025_ catalyst were well retained after CO_2_RR test (Figure S13, Supporting Information). Previous studies of Cu‐Sn bimetallic catalysts for CO_2_RR always exhibited high FEs for CO or formate at potentials higher than −1.1 V_RHE_ (Table S2, Supporting Information).^[^ ^18^, ^19^ ^]^ Herein, it is found that C_2_H_4_ activity is suppressed while C_2_H_5_OH production is boosted at potentials that is more negative than −1.1 V_RHE_ on our CuSn*_x_* catalysts. To validate the C_2_ products were generated from CO_2_RR, we then performed the electrolysis on CuSn_0.025_ catalyst under continuous bubbling of argon instead of CO_2_ and the result showed that C_2_H_4_ and C_2_H_5_OH were produced from CO_2_ reduction on CuSn_0.025_ catalyst (Figure S14, Supporting Information).

### 2.3 Selectivity Bifurcation and Surface Oxophilicity Characterization

**Figure 3:**
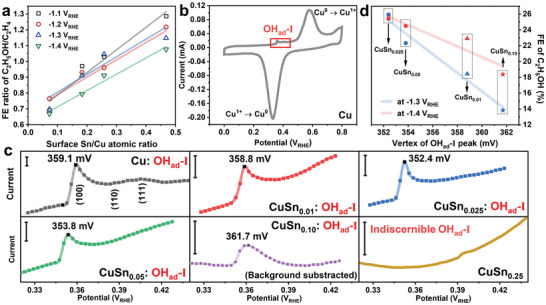
Selectivity correlation and surface oxophilicity characterization. a) The correlation between surface Sn/Cu atomic ratio and FE ratio of C_2_H_5_OH/C_2_H_4_. b) CV curves of bare Cu catalyst. c) Enlarged CV curves showing OH_ad_‐I adsorption peaks for Cu and CuSn*_x_* catalysts (The rulers represent 2 µA in each figure). d) The linear correlation of the oxophilicity of Cu sites with FEs of C_2_H_5_OH at −1.3 and −1.4 V_RHE_.

To gain further insight into the selectivity trend of C_2_H_4_ and C_2_H_5_OH on CuSn*_x_* catalysts, the FE ratios of C_2_H_5_OH/C_2_H_4_ of different CuSn*_x_* catalysts were compared at the potentials ranging from −1.1 to −1.4 V_RHE_ (Figure S15, Supporting Information). Obviously, the FE ratios of C_2_H_5_OH/C_2_H_4_ of the CuSn*_x_* catalysts were higher than that of the bare Cu catalyst and showed good linearity with the surface Sn/Cu ratio (**Figure** **3**a), CuSn_0.25_ catalyst was not included since the deviation is relatively large, which is likely due to its low FEs for both C_2_H_4_ and C_2_H_5_OH). It has been shown that the introduction of Sn into Cu significantly improved the production of CO in the whole potential range, which is believed to be the key intermediate for C—C coupling. The enhancement of local CO concentration could facilitate C_2+_ production via tandem effect on bimetallic catalysts.^[^ ^25^ ^]^ However, the reversed selectivity tendencies of C_2_H_4_ and C_2_H_5_OH in this work ruled out the tandem effect in our CuSn*_x_* catalysts. The surface oxophilicity of catalysts is of great importance for the processes that involves oxygen relevant species, such as *O, *OH, and adsorbed oxygenic intermediates.^[^ ^17^, ^26^ ^]^ Therefore, the binding strength of oxygen‐containing SDI of C_2_H_4_ and C_2_H_5_OH is expected to be influenced by the oxophilicity of the binding sites.^[^ ^14^, ^16^ ^]^ To this end, we first tried to understand how the surface oxophilicity changed with the component of the CuSn*_x_* catalysts. To accomplish that, cyclic voltammetry (CV) tests in 0.1 m NaOH solution were performed to determine the binding strength of adsorbed hydroxide ions (OH_ad_) on the CuSn*_x_* catalysts, which reflects their oxophilicity.^[^ ^26^, ^27^ ^]^ As depicted in Figure 3b and Figure S16 (Supporting Information), the CV curve of bare Cu and CuSn*_x_* catalyst showed Cu^0^—Cu^1+^ redox peaks at around 328 and 577 mV_RHE_.^[^ ^28^ ^]^ In addition, a peak associated with the adsorption of OH_ad_ appeared at around 360 mV_RHE_ (OH_ad_‐I).^[^ ^28^ ^]^ The zoomed‐in OH_ad_‐I peaks on bare Cu catalyst exhibited typical OH_ad_ peaks obtained on polycrystalline Cu, where a strong OH_ad_ peak corresponding to Cu(100) is at 359.1 mV_RHE_, followed by two weak OH_ad_ peaks assigned to Cu(110) and Cu(111) (Figure 3c).^[^ ^29^ ^]^ The introduction of Sn altered the adsorption behavior of OH_ad_‐I on Cu sites. On the CuSn*_x_* catalyst, there was only a strong OH_ad_‐I peak that shifted to more negative potentials, indicating the enhanced adsorption strength of OH_ad_ (Figure 3c).^[^ ^26b^ ^]^ The adsorption strength of OH_ad_‐I reached the maximum on CuSn_0.025_ catalyst, on which the onset and vertex of OH_ad_‐I peak negatively shifted to 343.1 and 352.4 mV_RHE_, indicating the strongest oxophilicity of Cu sites on CuSn_0.025_ (Figure 3c).^[^ ^29a^ ^]^ With the increasing Sn content in the CuSn*_x_* catalysts, the intensity of OH_ad_‐I peak decreased and an additional broad peak at around 150 mV_RHE_ (OH_ad_‐II) appeared on CuSn_0.05_ and CuSn_0.10_ catalysts (Figure 3c). Given the stronger O affinity of Sn than Cu, the intensified OH_ad_‐II peak with the increasing Sn content should be assigned to the strong adsorption of OH_ad_ sites of Sn. For CuSn_0.25_ catalyst, only a broad peak at 100–200 mV_RHE_ could be observed, indicating that strong adsorption of OH_ad_ on Sn sites prevailed when the surface is abundant in Sn (Figure 3c). These results demonstrate that the introduction of Sn on Cu surface enhances the oxophilicity of the CuSn*_x_* catalysts in two ways: 1) enhanced oxophilicity of Cu sites; 2) creation of new stronger oxophilic Sn sites. Based on the above results, we tried to elucidate the importance of surface oxophilicity to C_2_H_5_OH production in CO_2_RR. The FEs of C_2_H_5_OH at −1.3 and −1.4 V_RHE_ on the CuSn*_x_* catalysts were plotted against the vertex potentials of OH_ad_‐I peaks in the CV curves, which reflects the surface oxophilicity of the CuSn*_x_* catalysts (Figure 3d). The great linearity strongly supported the positive correlation between the oxophilicity of the Cu sites and C_2_H_5_OH production (bare Cu and CuSn_0.25_ catalysts are not plotted in Figure 3d because of the multiple OH_ad_‐I peaks in bare Cu catalyst and the indiscernible OH_ad_‐I peak in CuSn_0.25_ catalyst).

**Figure 4:**
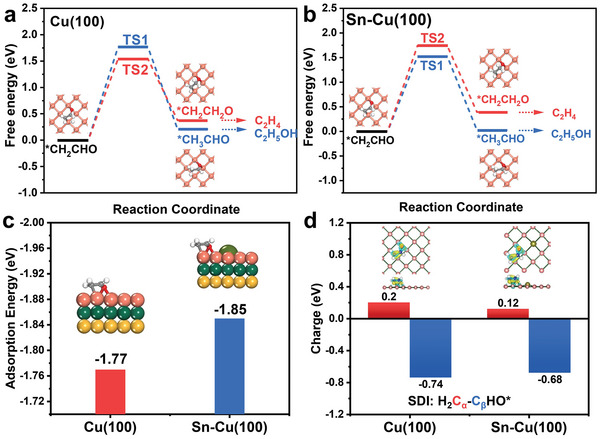
Theoretical study. The free energy diagrams of *CH_2_CHO reduction to *CH_2_CH_2_O or *CH_3_CHO on a) Cu (100) surface and b) Sn‐Cu(100) surface. c) The adsorption energy of *CH_2_CHO intermediate on Cu (100) and Sn‐Cu(100) surfaces. d) The calculated charge carried by C_*α*_ and C_*β*_ in adsorbed *CH_2_CHO intermediate on Cu (100) and Sn‐Cu(100) surfaces.

We have then performed theoretical calculations to understand the mechanistic insight into the unusual C_2_ products selectivity and the effects of oxophilicity. Previously, it has been evidenced by experimental and theoretical results that C_2_H_4_ and C_2_H_5_OH products branch after C—C coupling, namely, the SDI that bifurcates C_2_H_4_ and C_2_H_5_OH pathway is a C_2_ intermediate.^[^ ^2^, ^12^, ^30^ ^]^ Koper and co‐workers have previously shown that H_2_C—CHO* is the SDI that bifurcating C_2_H_4_ and C_2_H_5_OH.^[^ ^12b^ ^]^ The hydrogenation of C_*α*_ in H_2_C—CHO* leads to H_3_C—CHO*, which is further reduced to C_2_H_5_OH. On the contrary, the hydrogenation of C_*β*_ forms H_2_C—CH_2_O*, which leads to the formation of C_2_H_4_.^[^ ^7^, ^13^ ^]^ Based on these previous studies, we carried out theoretical calculation of the Gibbs free energy of these two pathways. Given the highly dispersed Sn into the Cu lattice in CuSn*_x_* catalysts and the high C_2_ selectivity of Cu(100) facet, slab models of Sn substituted Cu(100) facets were built and Sn‐Cu(100) slab was selected for free energy calculation (Figure S17, Supporting Information). As seen in **Figure** **4**a,b, on Cu(100), the energy barriers for C_2_H_4_ and C_2_H_5_OH pathways were 1.54 and 1.77 eV, respectively, suggesting the C_2_H_4_ formation is thermodynamically more favorable. In contrast, the energy barrier of C_2_H_5_OH pathway was 0.22 eV lower than that of C_2_H_4_ pathway on Sn‐Cu(100). The calculation results revealed that the C_2_H_5_OH pathway on Cu sites was enhanced by Sn modification, which is in accordance with the FEs trends. We then sought theoretical calculation for understanding the effects of oxophilicity on the SDI. As seen in Figure 4c, the stable SDI bonded to Cu sites on both Cu(100) and Sn‐Cu(100) and the calculated adsorption energy were −1.77 and −1.85 eV respectively, indicating the enhanced oxophilicity of Cu sites could stabilize the adsorption of SDI. As discussed above, the next proton and electron transfer step of the SDI bifurcates the C_2_H_4_ and C_2_H_5_OH pathways. The charge analysis of adsorbed SDI depicted in Figure 4d showed that the C_*α*_ carried less positive charge and the C_*β*_ also carried less negative charge on Sn‐Cu(100), which both contribute the next protonation on C_*β*_ to be easier on Sn‐Cu(100). Therefore, we believe that the enhanced oxophilicity of Cu sites in CuSn*_x_* catalysts stabilize the SDI and affect its protonation to facilitate the C_2_H_5_OH pathway in CO_2_RR.

**Figure 5:**
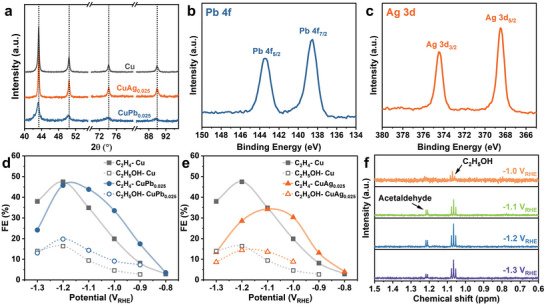
Confirmation of oxophilicity regulation strategy. a) XRD patterns of Cu, CuAg_0.025_, and CuPb_0.025_ catalysts. b) Pb 4f XPS spectrum of CuPb_0.025_ catalyst. c) Ag 3d XPS spectrum of CuAg_0.025_ catalyst. d,e) Comparison of FE of C_2_ products. f) ^1^H NMR patterns showing the peaks of multicarbon liquid products for CuAg_0.025_ catalyst.

To further verify the effect of surface oxophilicity on CO_2_RR, we combined Cu with Pb and Ag due to their suitable oxophilicity (Figure S1, Supporting Information, Ag < Cu < Pb < Sn) and redox property. Thus, CuPb_0.025_ and CuAg_0.025_ catalysts were prepared by the same method and their CO_2_RR performance was tested at −0.7 to −1.3 V_RHE_ (Figure S18, Supporting Information). Both catalysts exhibited XRD patterns resembled to bare Cu catalyst and the existence of Pb and Ag were demonstrated by XPS spectra (**Figure** **5**a–c). The FE of C_2_H_5_OH on CuPb_0.025_ catalyst was higher than bare Cu catalyst but lower than CuSn_0.025_ catalyst (Figure 5d), which is consistent with the oxophilicity trend. Moreover, the FEs of C_2_H_4_ for CuPb_0.025_ was comparable or even superior to bare Cu catalyst and the maximum FEs of C_2+_ for CuPb_0.025_ reached 70.78% at −1.2 V_RHE_. On the other hand, the FE of C_2_H_5_OH on CuAg_0.025_ catalyst was lower than that on bare Cu catalyst CuAg_0.025_ (Figure 5e), which agrees with the lower oxophilicity of Ag than Cu. These results strongly support the surface oxophilicity regulation strategy for improving C_2_H_5_OH production in CO_2_RR. Interestingly, there was no *n*‐C_3_H_7_OH product that could be detected on CuAg_0.025_ catalyst at any potentials, while a new doublet assigned to H in acetaldehyde emerged in the ^1^H NMR patterns (Figure 5f). Although the mechanism of the acetaldehyde formation is rarely reported, it is believed that acetaldehyde formed on the C_2_H_5_OH pathway.^[^ ^31^ ^]^ Such interesting selectivity behaviors of CuPb_0.025_ and CuAg_0.025_ catalysts imply that the effect of surface oxophilicity cannot be accomplished alone without other effects in bimetallic catalysts, such as electronic and strain effects.^[^ ^8^, ^11^ ^]^ It should be noted that the FE of C_2_H_5_OH is still relatively low even at high overpotential on CuSn*_x_* catalyst, which undermines its application prospect. However, we would like to stress the regulation of selectivity between C_2_ products by surface oxophilicity engineering and further work is needed to distinguish surface oxophilicity from other effects in bimetallic systems. Besides, it is believed that this strategy can cooperate with other effects that facilitate the C—C coupling, such as tandem effect and Cu^1^—Cu^0^ synergistic effect,^[^ ^3^, ^25^, ^32^ ^]^ to further increase the production of C_2+_ oxygenic products in CO_2_RR.

## 3 Conclusion

In summary, we have proposed the surface oxophilicity regulation strategy to improve C_2_H_5_OH production in CO_2_RR via steering the binding strength of the adsorbed intermediate. A series of CuSn*_x_* bimetallic catalysts featured typical dendrite microstructure with surface‐enriched Sn species dispersed in Cu matrix were synthesized. The CuSn*_x_* catalysts exhibited enhanced CO_2_RR to 2e products at low and moderate overpotentials, while high FE of C_2_H_5_OH and increased C_2_H_5_OH/C_2_H_4_ ratio were achieved at large overpotentials. It was found that the introduction of Sn atom on Cu surface could enhance the surface oxophilicity of the catalysts, which play a key role in bifurcating the reaction pathways between C_2_H_4_ and C_2_H_5_OH by steering the protonation of the key oxygenic intermediate. Based on the above experimental and theoretical results, we believe that the surface oxophilicity regulation of Cu‐based catalysts is a promising strategy to design highly efficient electrocatalysts for oxygenic products in CO_2_RR.

## 4 Experimental Section

### Materials Synthesis

First, a piece of Zn foil (1 cm × 1 cm × 0.1 mm) was sonicated in water, ethanol, and acetone for 30 min, respectively. Then 10 mL of mixed metal ions solution containing certain amount of CuCl_2_∙2H_2_O and SnCl_2_∙2H_2_O was mixed in 100 × 10^−3^ m HCl. The total concentration of metal ions was 100 × 10^−3^ m and the metal content was controlled by varying the concentration of CuCl_2_∙2H_2_O and SnCl_2_∙2H_2_O. Then the cleaned Zn foil was immersed in 0.1 m HCl for 5 min to remove the surface oxide layer before immersing into above solution. After 1 h of replacement reaction at room temperature, the reaction was stopped by pouring out the solution. To remove the surplus Zn metal completely, the Cu‐Sn dendrite deposition was then washed with diluted HCl solution (0.1 m) and then washed with ethanol three times by centrifugation. The obtained material was dried under vacuum and denoted as CuSn*_x_* catalysts, where *x* stands for the Sn percentage in the metal ion solution. For instance, CuSn_0.025_ catalyst was prepared by replacement reaction between Zn foil and 10 mL metal ion solution containing 2.5 × 10^−3^ m SnCl_2_, 97.5 × 10^−3^ m CuCl_2_, and 100 × 10^−3^ m HCl. The CuSn*_x_* catalysts were stored under inert atmosphere in a glove box (O_2_ < 0.1 ppm, H_2_O < 0.1 ppm). It was found that trace amount of Zn species remained in all CuSn*_x_* catalysts, which is likely due to the surface attached Zn^2+^ ions. As determined by XPS measurements, the Zn content in CuSn*_x_* catalysts ranged from 0.6% to 1.1% (Table S1, Supporting Information), which is far less than the Cu and Sn content in CuSn*_x_*. Therefore, the effect of residual Zn in CuSn*_x_* catalysts is not considered in this work.

CuPb_0.025_ and CuAg_0.025_ were synthesized by the similar method to CuSn_0.025_. The metal ions solution for the synthesis of CuPb_0.025_ contains 100 × 10^−3^ m of Cu(NO_3_)_2_∙3H_2_O and Pb(CH_3_COO)_2_. For the synthesis of CuAg_0.025_, ammonia water was added drop by drop into a solution of 10 mL containing 0.975 mmol Cu(NO_3_)_2_∙3H_2_O and 0.025 mmol AgNO_3_ under ultrasonic until the initially formed precipitate was just dissolved, forming a dark blue solution. Then 100 mg Mg powder was added into this solution and left still for 1 h. The obtained solid was filtered and washed with 0.1 m HCl solution until no bubbles appeared. Finally, the product was washed with deionized water for five times and dried under vacuum at room temperature.

### Electrode Preparation

1 mg of as‐prepared CuSn*_x_* catalysts and 0.2 mg of carbon black were dispersed in 190 *µ*L methanol. Subsequently, 10 *µ*L of Nafion (5 wt.%) was added, followed by ultrasonication for at least 1 h. Then 6 *µ*L catalyst ink was dropped onto a L‐type glass carbon electrode with a diameter of 4 mm (geometric area ≈ 0.1256 cm^2^) using a pipette and dried under ambient air. The catalyst loading was about 0.24 mg cm^−2^. The catalysts were electrochemically activated in CO_2_‐saturated 0.1 m KHCO_3_ solution by CV (ten scans) from −0.5 to −1.5 V_RHE_. The addition of carbon black in preparation of catalyst ink is to enhance the conductivity of catalyst layer on the electrode. To exclude the effect of carbon black on the CO_2_RR performance, CO_2_RR test on carbon black (without CuSn*_x_* catalysts) was carried out under identical CO_2_RR test conditions at −1.2 V_RHE_. As shown in Figure S19 (Supporting Information), the carbon black exhibited fairly low current density with nearly no CO_2_RR activity, demonstrating that the addition of carbon black has little effect on the CO_2_RR performance.

### Electrochemical Measurements

CO_2_ electrolysis was carried out in a gas‐tight, custom‐made two‐compartment cell, in which the working electrode was separated from the counter electrode by a Nafion 117 membrane. Ag/AgCl (saturated KCl) was used as the reference electrode and the three‐electrode setup was connected to a potentiostat (Biologic VMP3). A 0.1 m KHCO_3_ electrolyte solution was used for all CO_2_RR tests and the electrolyte in the cathodic compartment was stirred at a constant rate of 500 rpm during electrolysis. Before CO_2_RR, the catholyte were bubbled with CO_2_ (99.99%) for 20–30 min to reach saturation, and CO_2_ was kept purging into the cathodic compartment at flowrate of 20 sccm during the CO_2_RR. To determine the FEs of the reduced products, chronoamperometry was performed for 1 h at *iR*‐corrected potentials. The typical CA curves of CuSn_0.025_ recorded during the CO_2_RR tests at different potentials are presented in Figure S13a (Supporting Information).

All potentials were measured against an Ag/AgCl reference electrode (saturated KCl) and converted to the reversible hydrogen electrode (RHE) scale by

(1) $$E_{\mathrm{RHE}}=E_{\mathrm{Ag}/\mathrm{AgCl}}\;+0.198+0.059\times \mathrm{pH}$$

All the potentials in the text were *iR*‐corrected. The resistance between the reference and working electrodes was measured by potential electrochemical impedance spectroscopy (PEIS) and 80% of the ohmic drop was compensated automatically by software and the rest 20% remained uncompensated. Generally, the value of uncompensated resistance (Ru) determined by PEIS was about 110–115 Ω in CO_2_ saturated 0.1 m KHCO_3_ in this work.

The CV tests were carried out in Ar‐saturated 0.1 m NaOH solution. The working electrode was prepared by the same method as for the chronoamperometry tests and electrochemically activated in CO_2_‐saturated 0.1 m KHCO_3_ solution by CV (ten scans) from −0.5 to −1.5 V_RHE_ before CV tests in alkaline solution. The scan rate was 10 mV s^−1^ and the potential range was 0–0.8 V_RHE_ (−0.2 to 0.8 V_RHE_ for CuSn_0.25_).

### Materials Characterization

SEM images were obtained using field emission scanning electron microscope of TESCAN MAIA3 with EDS of Bruker Quantax 200 XFlash. TEM images were acquired using FEI Talos F200S. HAADF‐STEM and elemental mapping analysis were performed on FEI Talos F200S. Powder XRD was obtained using Bruker D2 Phaser. XPS measurements were performed on Escalab 250Xi and Ar sputtering was carried out at 1000 eV for 300 s. ^1^H NMR spectra were obtained on Bruker AVANCE III 600 MHz nuclear magnetic resonance spectrometer.

### Calculation Details

The Vienna ab initio simulation package (VASP) code was used for DFT calculations in this work with projected augmented wave (PAW) pseudopotential to approximate the interaction between ion cores and valence electrons. A plane wave cutoff energy of 400 eV was chosen, together with Perdew–Burke–Ernzerhof (PBE) method as the exchange‐correlation functional. A Monkhorst–Pack mesh of 5 × 5 × 1 k‐points was used for the Brillouin zone integration. A force convergence of 0.03 eV Å^−1^ was used in geometry optimization.

The optimum lattice parameters of the Cu unit cell were calculated to be *a* = 3.631 Å, close to the experimental values of 3.615 Å. The Cu(100) surface was modeled using a p (3 × 3) supercell with five‐layer slab, the Cu(110) surface was modeled using a p (3 × 2) supercell with seven‐layer slab, and the Cu(111) surface was modeled using a p (2 × 2) supercell with five‐layer slab. The bottom two layers were fixed at the bulk lattice position. A 15 Å vacuum layer was used to separate the surface from the periodic image in the direction along the surface of the slab. The energy of adsorption (*E*_ads_) was calculated using *E*_ads_ = *E*_complex_ − (*E*_adsorbate_ + *E*_surface_), where *E*_complex_ represents the energy of adsorbate on the Cu(100) surface, *E*_adsorbate_ and *E*_surface_ are the energies of isolated adsorbate and on Cu(100) surface, respectively.

### Gas Product Analysis

Gas products from the cathodic compartment during CO_2_RR were analyzed using a GC‐2014 (Shimadzu) equipped with a TCD detector and two FID detectors, one of which was coupled with a methanizer to detect low concentration of CO. High purity Ar (99.999%) was used as the carrier gas. The gas products, including H_2_, CO, CH_4_, and C_2_H_4_ were calibrated using standard mixed gases with different content (Dalian Special Gases Co., Ltd.) and the calibration curves were shown in Figure S20 (Supporting Information).

The Faradaic efficiencies of the gas products were calculated by the GC data using the following equation

(2) $${\mathrm{FE}}_{g}=\frac{Q_{g}}{Q_{\mathrm{total}}}\;\times 100\%\;=\frac{\frac{v}{60\;s/\min}\times \frac{y}{24.5\;L/\mathrm{mol}}\times n\times F}{j_{\mathrm{average}}}\;\times 100\%$$

where *v* is gas flow rate measured by a flowmeter, which is 10.0 sccm for all the tests. *y* is the measured volumetric content of the gas product. *n* is the number of electrons required to form the gas products, and *n* = 2, 2, 8, and 12 for H_2_, CO, CH_4_, and C_2_H_4_, respectively. *F* is the Faraday constant (96 485 C mol^−1^). *j* is the average current density.

### Liquid Product Analysis

Liquid products were analyzed by a 600 MHz NMR spectrometer (Bruker Avance 3 HD 600 MHz) using a presaturation technique to suppress water peak. To perform ^1^H NMR measurement, 800 *µ*L of electrolyte sampled after CA tests was mixed with 100 *µ*L DMSO standard solution (100 ppm) and 100 *µ*L D_2_O. Figure S20 (Supporting Information) showed the calibration curves of the liquid products, which was plotted by measuring standard solutions containing possible liquid products, including formate, acetate acid, methanol, ethanol, and 1‐propanol. The faradaic efficiencies of liquid products were calculated as follows

(3) $${\mathrm{FE}}_{l}=\frac{Q_{l}}{Q_{\mathrm{total}}}\;\times 100\%\;=\frac{n_{l}\times n\times F}{Q_{\mathrm{total}}}\;\times 100\%$$

where *n*_l_ is the total content of certain liquid products in the catholyte, which was calculated by the concentration and the volume of the catholyte (20 mL). *n* is the number of electrons required to form the liquid products, and *n* = 2, 6, 8, 12, and 18 for formate, methanol, acetate, ethanol, and 1‐propanol, respectively.

## Conflict of Interest

The authors declare no conflict of interest.

## Supporting information

Supporting Information

## Data Availability

The data that support the findings of this study are available on request from the corresponding author. The data are not publicly available due to privacy or ethical restrictions.
