# Supplementary material for: Engineering Surface Oxophilicity of Copper for Electrochemical CO2 Reduction to Ethanol
Source: Adv Sci (Weinh). 2022 Nov 17;10(2):2204579. doi: 10.1002/advs.202204579 (PMC9839838; doi:10.1002/advs.202204579)
Supplement: Supplementary file 1 — Supporting Information [file ADVS-10-2204579-s001.pdf]

ADVANCED SCIENCE
Open Access

Supporting Information

for *Adv. Sci.*, DOI 10.1002/advs.202204579

Engineering Surface Oxophilicity of Copper for Electrochemical CO_2 Reduction to Ethanol

*Minhan Li, Nan Song, Wei Luo, Jun Chen, Wan Jiang and Jianping Yang**

((Supporting Information can be included here using this template))

Supporting Information

Engineering Surface Oxophilicity of Copper for Electrochemical CO_2 Reduction to Ethanol

Minhan Li, Nan Song, Wei Luo, Jun Chen, Wan Jiang, Jianping Yang*

M. H. Li, W. Luo, W. Jiang, and J. P. Yang
State Key Laboratory for Modification of Chemical Fibers and Polymer Materials, College of Materials Science and Engineering, Donghua University, Shanghai 201620, P. R. China
E-mail: jianpingyang@dhu.edu.cn
N. Song
State Key Laboratory of Chemical Engineering, East China University of Science and Technology, Shanghai, 200237, P. R. China
J. Chen
ARC Centre of Excellence for Electromaterials Science, Intelligent Polymer Research Institute, Australian Institute of Innovative Materials, University of Wollongong, Innovation Campus, Wollongong, NSW 2522, Australia
M. H. Li
College of Materials Science and Engineering, Zhengzhou University, Zhengzhou, 450001, P. R. China

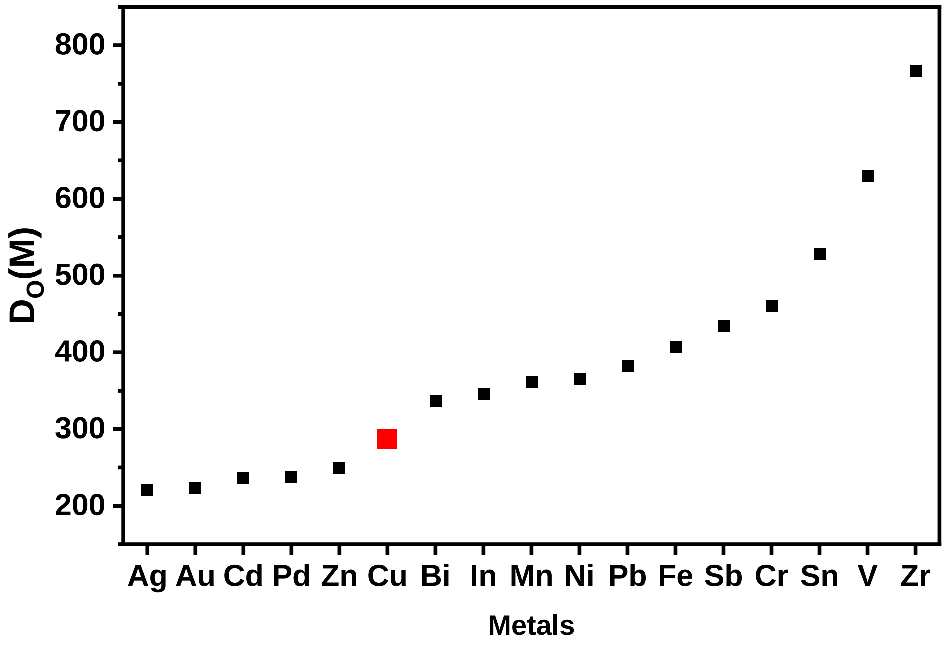

Figure S1 The oxophilicity of common transition metals and p-block metals determined by the bond dissociation enthalpies of metal oxides.[s1]

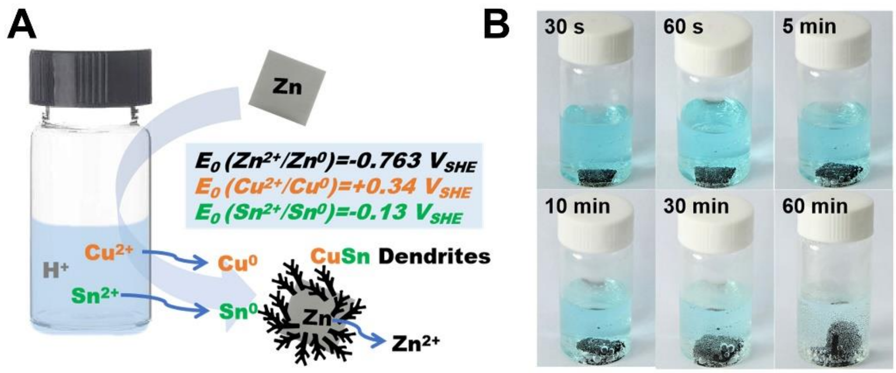

Figure S2 Schematic diagram of the materials synthesis (a). Photographs illustrate the synthesis procedures (b).

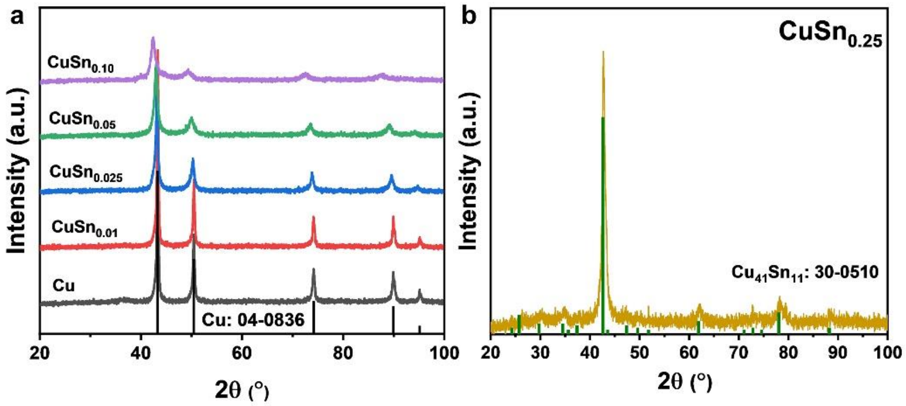

Figure S3 XRD pattern of Cu to $CuSn_{0.10}$ catalysts (a) and $CuSn_{0.25}$ catalyst (b).

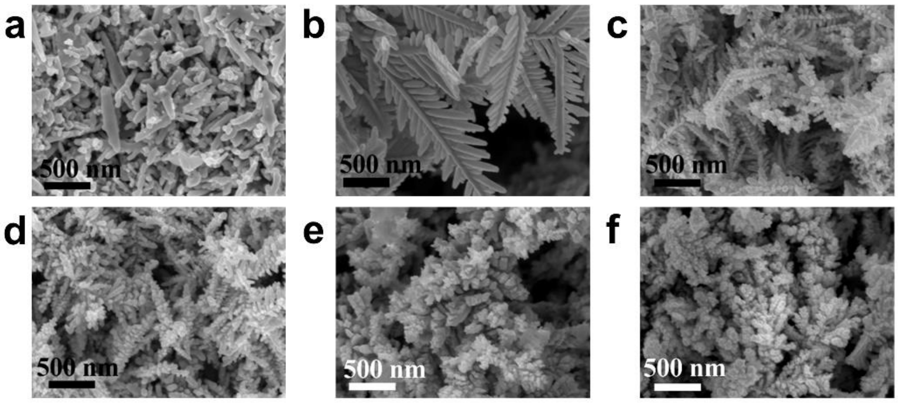

Figure S4 SEM images of (a) bare Cu, (b) $CuSn_{0.01}$, (c) $CuSn_{0.025}$, (d) $CuSn_{0.05}$, (e) $CuSn_{0.10}$, and (f) $CuSn_{0.25}$ catalysts.

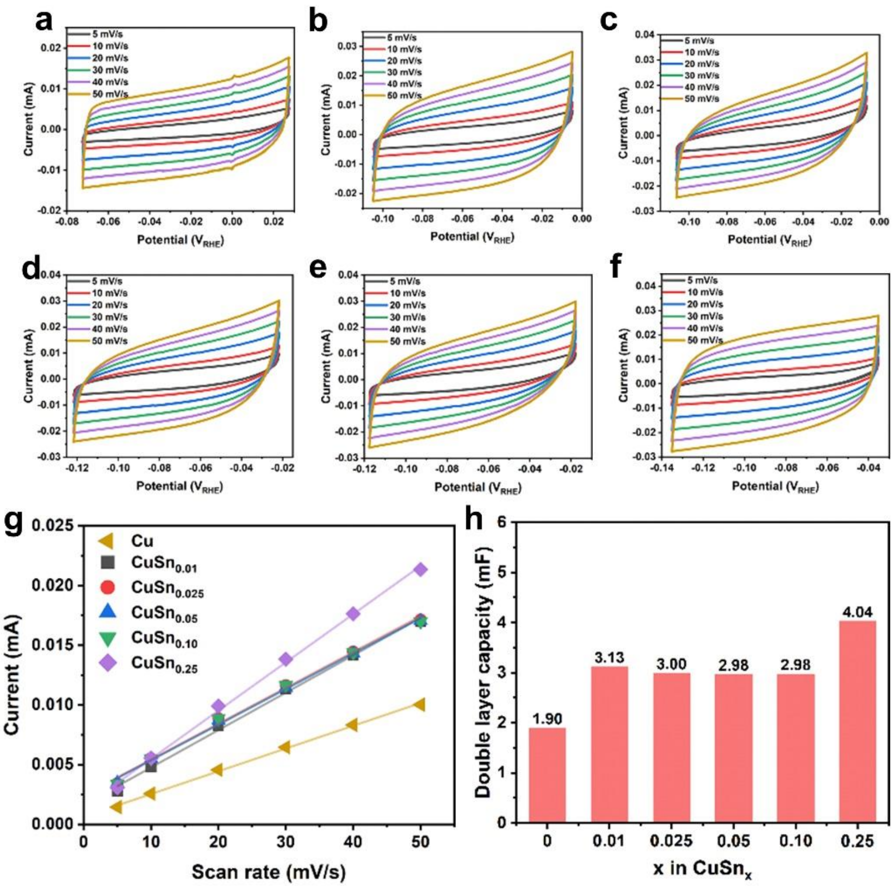

Figure S5 ECSA measurements of $CuSn_x$ catalysts. (a-f) Double layer capacities (C_{dl}) measured by CV curves in CO_2-saturated 0.1 M $KHCO_3$ solution at around open circuit potential (E_{OC} ± 50 mV) of Cu, $CuSn_{0.01}$, $CuSn_{0.025}$, $CuSn_{0.05}$, $CuSn_{0.10}$, and $CuSn_{0.25}$ catalysts. (g) Charge current density in CV scans plotted against the scan rates for $CuSn_x$ catalysts. (h) ECSA values of $CuSn_x$ catalysts.

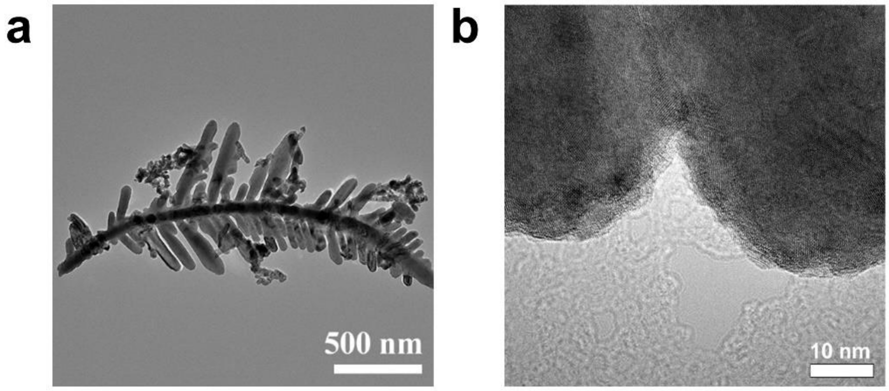

Figure S6 (a)TEM and (b) HR-TEM images of $CuSn_{0.01}$ catalyst.

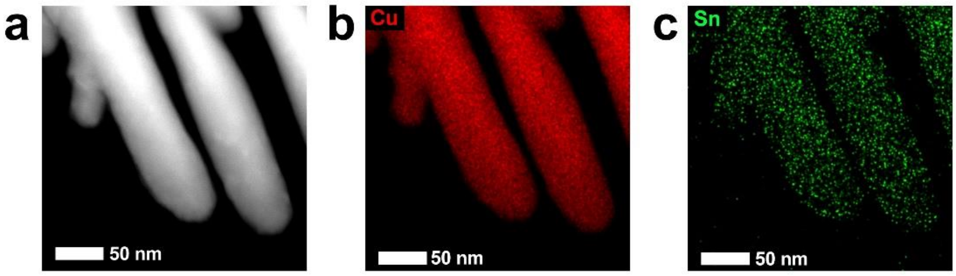

Figure S7 The HAADF-STEM image (a) and EDS element mappings of Cu (b) and Sn (c).

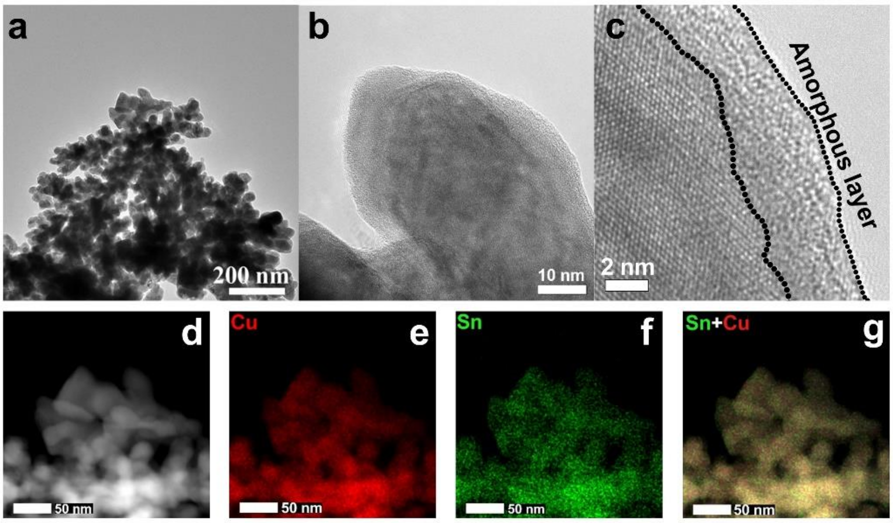

Figure S8 Characterization of $CuSn_{0.10}$ catalyst. (a) TEM image, (b-c) HR-TEM images, (d) HAADF-STEM image, and (e-g) EDS elemental mappings of $CuSn_{0.10}$ catalyst.

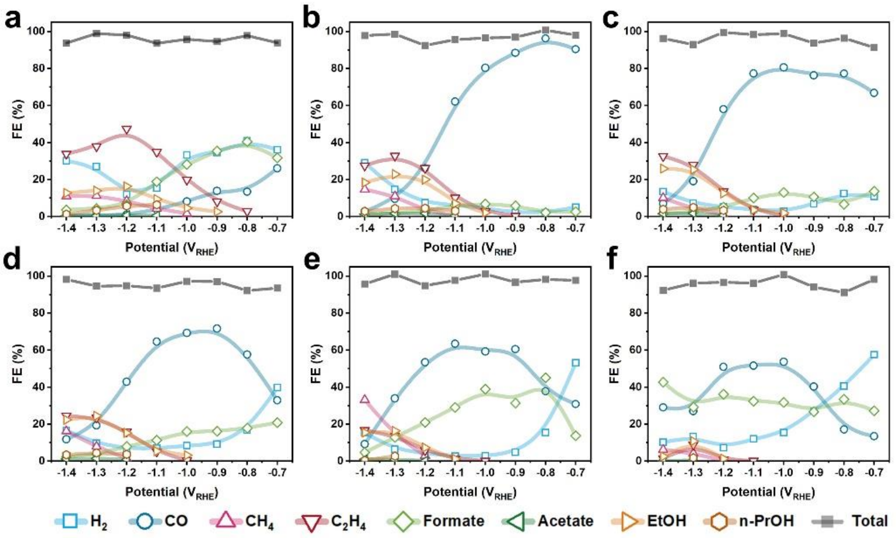

Figure S9 The distribution of reduction products at applied potentials on $CuSn_x$ catalysts. (a) Cu, (b) $CuSn_{0.01}$, (c) $CuSn_{0.025}$, (d) $CuSn_{0.05}$, (e) $CuSn_{0.10}$, and (f) $CuSn_{0.25}$.

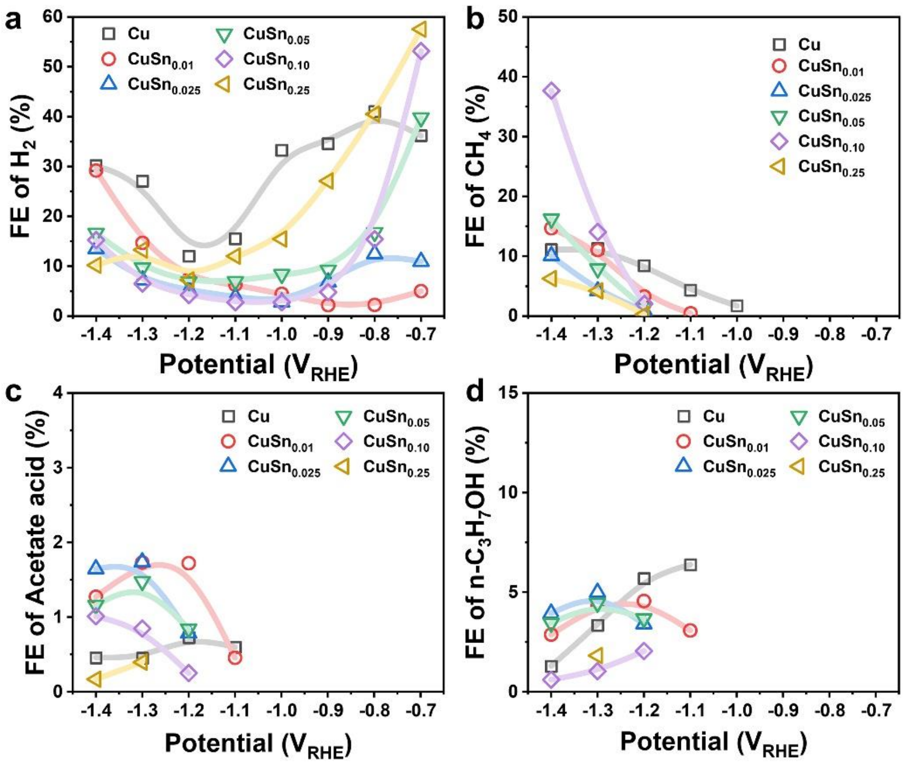

Figure S10 The FEs of reduction products. (a) H_2, (b) CH_4, (c) acetate acid, (d) n-C_3H_7OH.

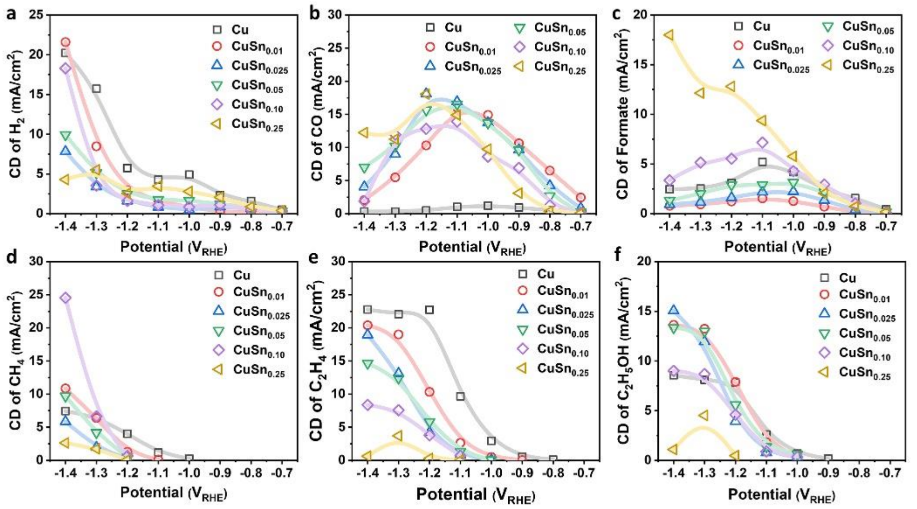

Figure S11 Current density (CD) of the main reduction products. (a) H_2. (b) CO. (c) formate. (d) CH_4. (e) C_2H_4. (f) C_2H_5OH.

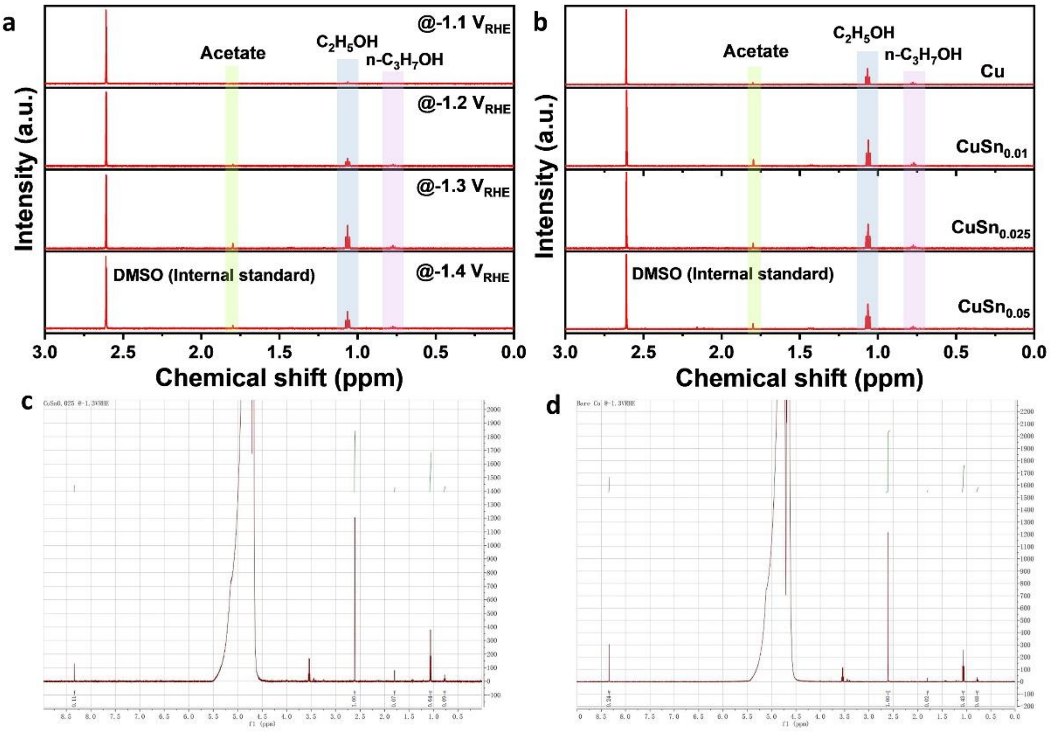

Figure S12 The typical ^{1}H NMR patterns that shows the peaks for multicarbon liquid products. (a) $CuSn_{0.025}$ catalysts at different potentials. (b) Different catalysts at -1.3 V_{RHE}. The original ^{1}H NMR patterns for liquid products determination of CO_2RR on $CuSn_{0.025}$ (c) and bare Cu catalyst (d) at -1.3 V_{RHE}.

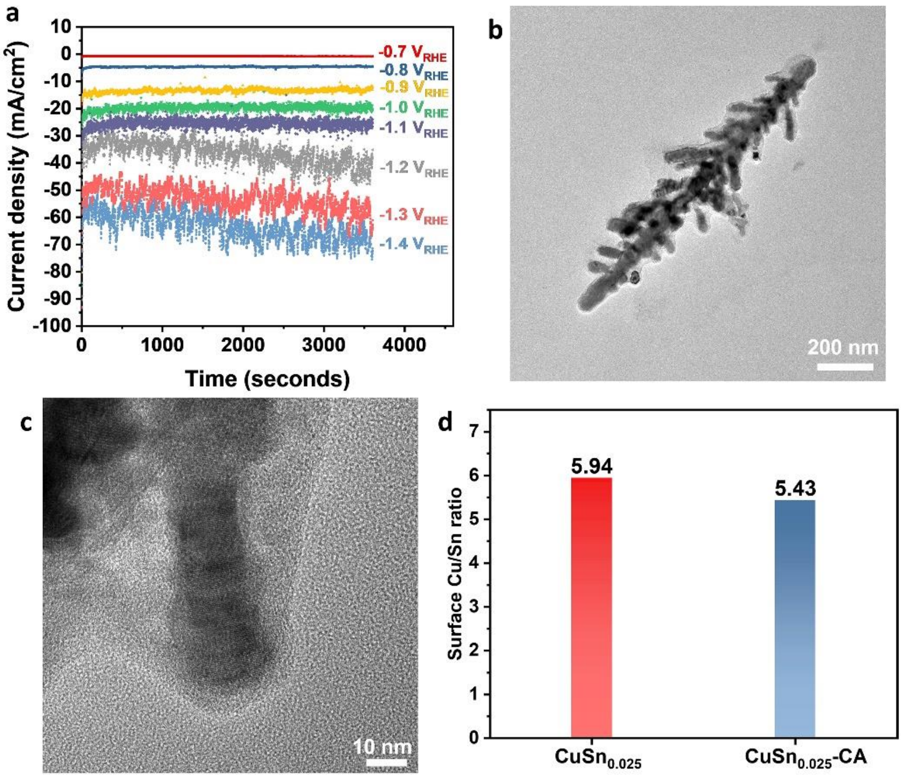

Figure S13 (a) The CA tests of $CuSn_{0.025}$ catalyst at different applied potentials. The current density was stable at different potentials, even though the current fluctuated at high overpotential due to the violent formation of bubble on the electrode. The TEM (b) and HR-TEM (c) images of $CuSn_{0.025}$ catalyst after CO_2RR test at -1.3 V_{RHE}. The dendrite morphology and crystalline structure were well retained at such high overpotential. The organic layer around the catalyst is the Nafion binder. (d) The surface Cu/Sn ratio of $CuSn_{0.025}$ catalyst determined by XPS measurement before and after chronoamperometry test ($CuSn_{0.025}$-CA).

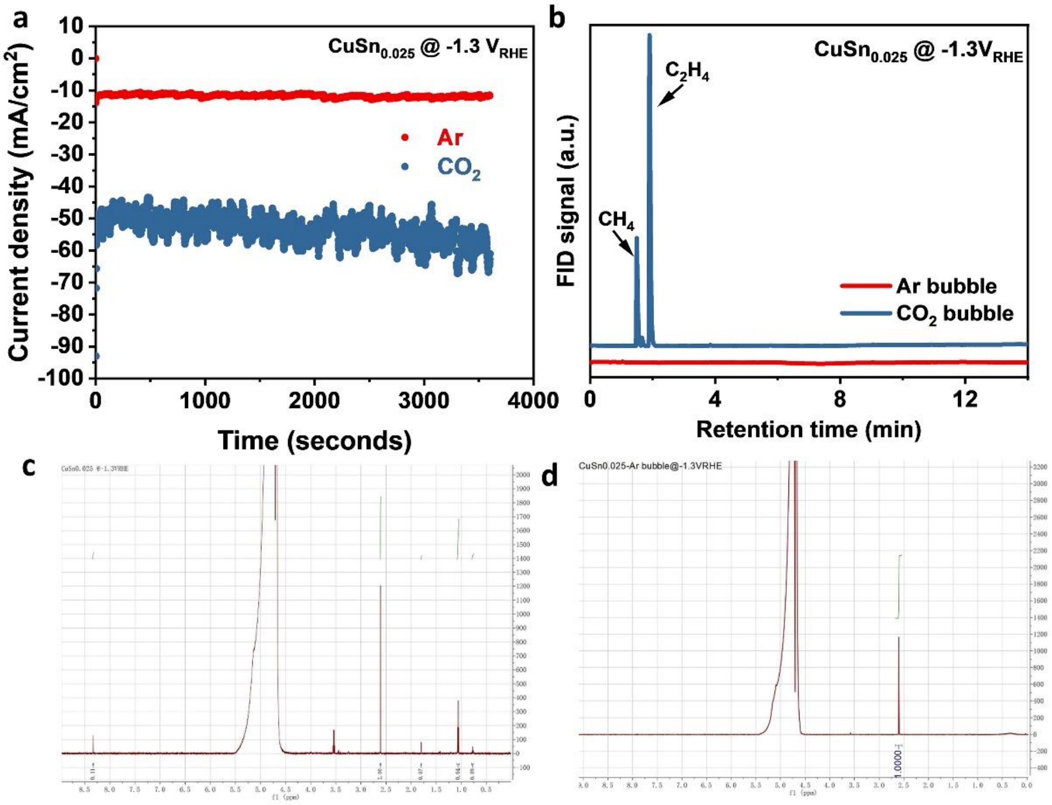

Figure S14 The comparison of electrolysis under CO_2 and Ar bubbling. (a) The chronoamperometry curves of $CuSn_{0.025}$ catalyst at -1.3 V_{RHE} under Ar and CO_2 bubbling. (b) The GC signal of the gaseous products of the electrolysis under CO_2 and Ar bubbling. (c-d) The ^{1}H NMR patterns of the liquid products after the electrolysis under CO_2 (c) and Ar (d) bubbling.

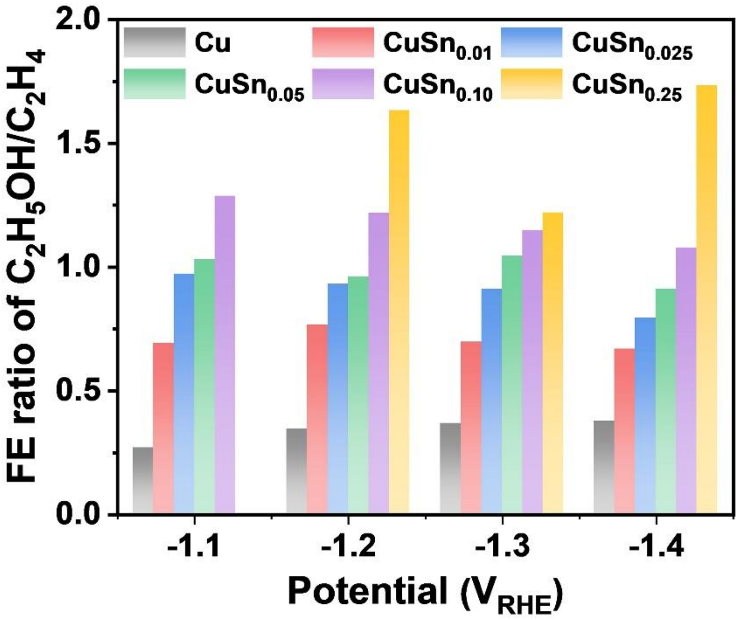

Figure S15 The FE ratio of C_2H_5OH/C_2H_4 on $CuSn_x$ catalysts at different potentials.

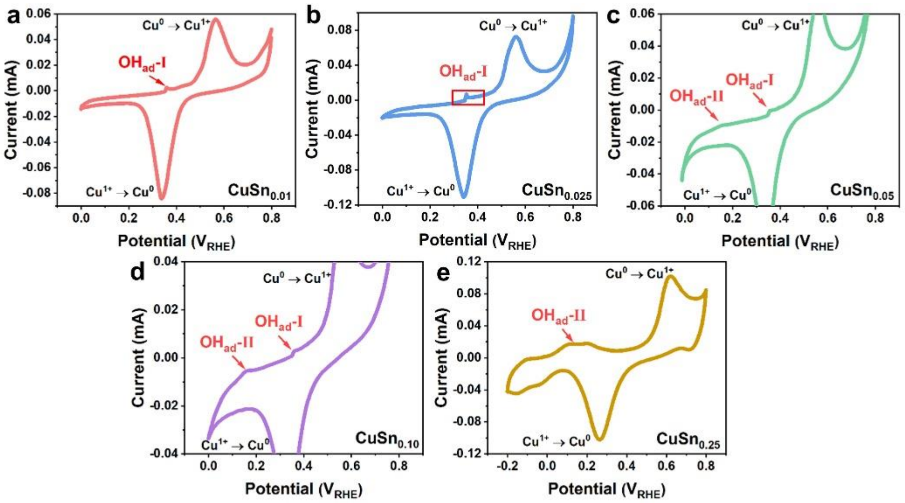

Figure S16 CV curves of (a) $CuSn_{0.01}$, (b) $CuSn_{0.025}$, (c) $CuSn_{0.05}$, (d) $CuSn_{0.10}$, and (e) $CuSn_{0.25}$ catalysts in 0.1 M NaOH.

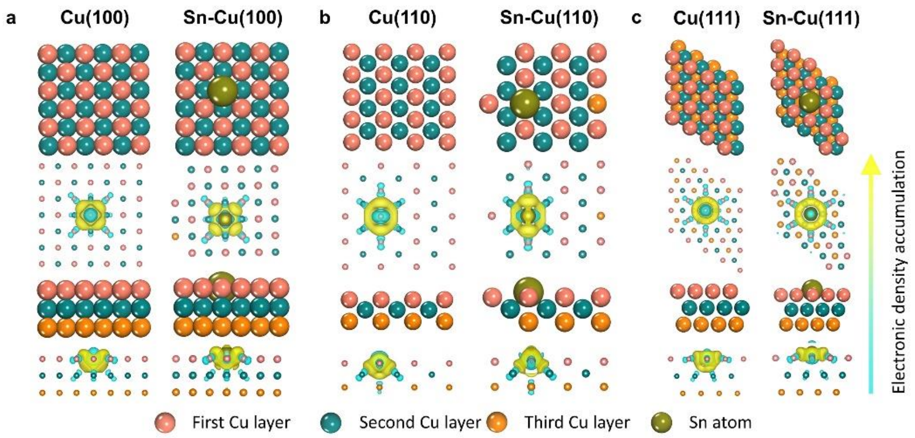

Figure S17 The slab models of Sn modified Cu facets for calculation and the corresponding differential charge analysis.

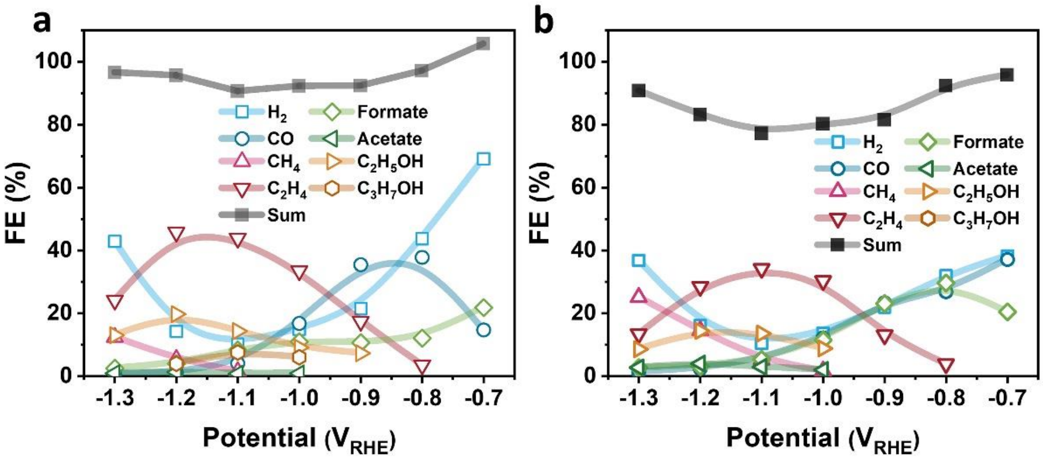

Figure S18 CO_2RR performance of $CuPb_{0.025}$ and $CuAg_{0.025}$ catalyst. (a) FEs of all products at different potentials for $CuPb_{0.025}$ catalyst. (b) FEs of all products at different potentials for $CuAg_{0.025}$ catalyst.

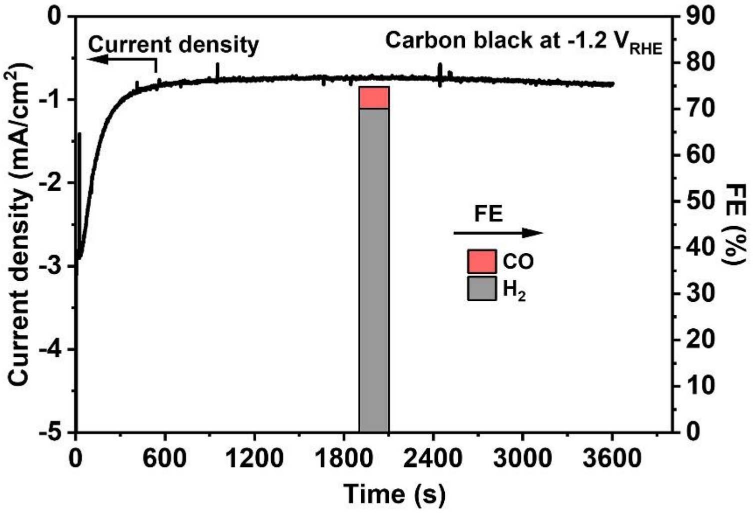

Figure S19 The blank CO_2RR test on carbon black catalyst at -1.2 V_{RHE}.

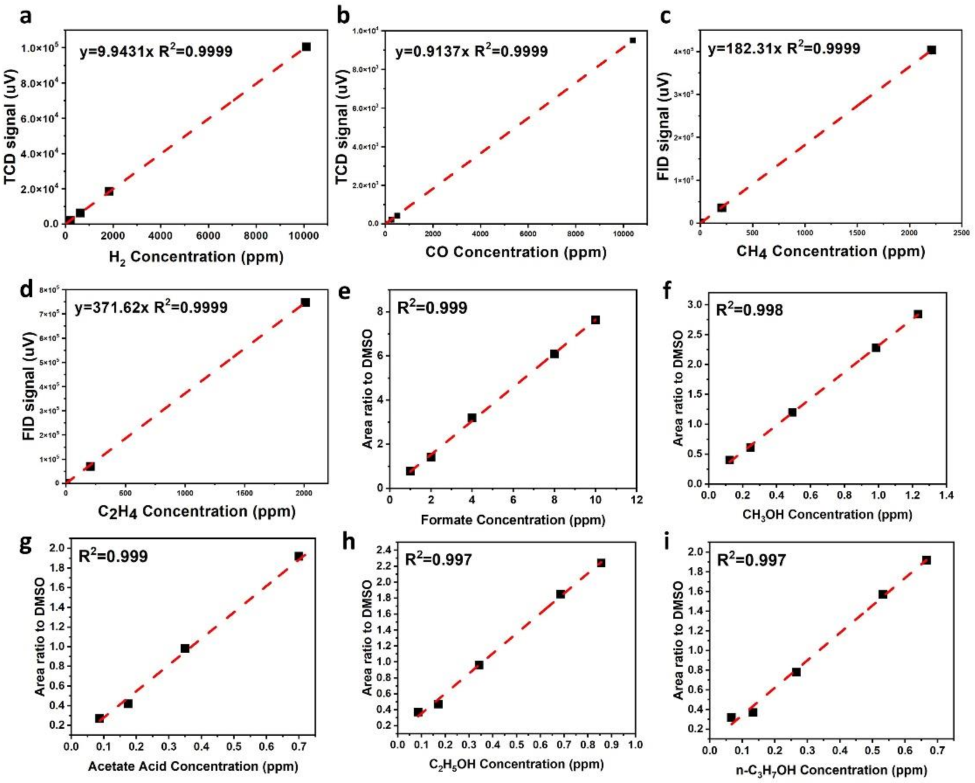

Figure S20 Gas and liquid products calibration. (a-d) Gas products. (e-i) Liquid products.

Table S1 ICP-AES results and residual Zn on the surface of $CuSn_x$ catalysts.

Catalyst	Cu concentration, ppm	Sn concentration, ppm	Cu:Sn weight percentage	Surface Zn percentage (XPS), at.%
Cu	12.6549	/	/	0.64
$CuSn_{0.01}$	22.0888	0.5557	39.75	0.86
$CuSn_{0.025}$	19.0830	1.3067	14.60	1.00
$CuSn_{0.05}$	16.4537	1.9974	8.24	1.12
$CuSn_{0.10}$	20.9182	5.7705	3.63	0.77
$CuSn_{0.25}$	16.0966	12.8509	1.25	0.97

Table S2 Summary of CO_2RR performance of the $CuSn_x$ catalysts in this work and the comparison of other Cu-Sn bimetallic catalysts in the literatures.

Catalyst	Main product	FE	Partial current density, mA/cm^2	Potential, V_{RHE}	Electrolyte	Reference
**$CuSn_{0.01}$**	**CO**	**96.36 %**	**6.51**	**-0.8**	**0.1 M $KHCO_3$**	**This work**
**$CuSn_{0.025}$**	**C_2H_5OH**	**25.93%**	**15.05**	**-1.4**	**0.1 M $KHCO_3$**	**This work**
Sn/Cu_2O	CO	87.9 %	~ 15 (total)	-1.3	0.1 M $KHCO_3$	S2
$Cu_{97}Sn_3$	CO	98 %	~ 30 (total)	-0.7	0.5 M $KHCO_3$	S3
CuSn40	CO	90.9 %	~ 3.3 (-0.8 V_{RHE})	-1.0	0.1 M $KHCO_3$	S4
Cu-Sn form	CO	93-94 %	6.2	-0.9	0.1 M $KHCO_3$	S5
Cu–Sn20	CO	82 %	0.96	-0.7	0.1 M $KHCO_3$	S6
Cu-Sn dendrite	CO	74 %	11.5	-1.1	0.1 M $KHCO_3$	S7
SnO_2/CuS NSs	CO	~70 %	15.24	-1.0	0.1 M $KHCO_3$	S8
Sn/Cu-PVDF	CO	80 %	104	< -0.9	0.1 M $KHCO_3$	S9
Cu−Sn	CO	90 %	1.0	-0.6	0.1 M $KHCO_3$	S10
C-Cu/SnO_2-0.8	CO	93 %	4.6	-0.7	0.5 M $KHCO_3$	S11
$Cu_{87}Sn_{13}$	CO	59.5 %	~ 0.9	-0.99	0.1 M $KHCO_3$	S12
CuO+SnO_2	CO	~ 90 %	~ 0.5	-0.6	0.1 M $NaHCO_3$	S13
Cu/SnO_x–CNT (6.2 % SnO_x)	CO	89 %	11.3	-0.99	0.1 M $KHCO_3$	S14
C-Cu/SnO_2-1.8	Formate	85 %	n.a.	-0.9	0.5 M $KHCO_3$	S11
$Cu_{55}Sn_{45}$	Formate	89.5 %	~ 2.5	-1.09	0.1 M $KHCO_3$	S12
Cu/SnO_x–CNT (30.2 % SnO_x)	Formate	77 %	4.0	-0.99	0.1 M $KHCO_3$	S14
$CuSn_{0.175}$-	CO+For	93 %	n.a.	-1.0	0.5 M	S15

NG	mate				$KHCO_3$	
Cu@Sn	Formate	~ 100 %	16.52	-0.93	0.5 M $KHCO_3$	S16
CuSn NPs/C-A	CO	70.1 %	1.66	-0.7	0.1 M $KHCO_3$	S17
	Formate	71.5 %	12.6	-1.0		
$Cu_{(1)}Sn_{(4)}$-N-CC	Formate	90.24 %	15.56	-0.97	0.5 M $KHCO_3$	S18
Sn-Cu alloy	Formate	82.3 %	79	-1.14	0.5 M KCl	S19
CuSn–10C	Formate	82 %	18.9	-1.0	0.1 M $NaHCO_3$	S20
CuSn NWs/C-Air	Formate	90.2 %	17.33	-1.0	0.5 M $KHCO_3$	S21
Cu_{oh}–Ag	C_2H_5OH	23.1 %	2.5	-1.4	0.1 M $KHCO_3$	S22
Cu(Ag-20)$_{20}$	C_2H_5OH	16.5 %	4.14	-1.1	0.1 M $KHCO_3$	S23
CuAg wire	C_2H_4	~60 %	~180	-0.7	1 M KOH	S24
CuZn Alloy	C_2H_4	33.3 %	6.1 (Total)	-1.1	0.1 M $KHCO_3$	S25

Reference

S1. Kepp, K.P. A quantitative scale of oxophilicity and thiophilicity. *Inorg. Chem.* **2016**, 55, 9461-9470.

S2. Zhang, W., He, P., Wang, C., Ding, T., Chen, T., Liu, X., Cao, L., Huang, T., Shen, X., Usoltsev, O.A., et al. Operando evidence of Cu^+ stabilization via a single-atom modifier for CO_2 electroreduction. *J. Mater. Chem. A* **2020**, 8, 25970-25977.

S3. Ren, W., Tan, X., Qu, J., Li, S., Li, J., Liu, X., Ringer, S.P., Cairney, J.M., Wang, K., Smith, S.C., and Zhao, C. Isolated copper–tin atomic interfaces tuning electrocatalytic CO_2 conversion. *Nat. Commun.* **2021**, 12, 1449.

S4. Zhang, S.-N., Li, M., Hua, B., Duan, N., Ding, S., Bergens, S., Shankar, K., and Luo, J.-L. A rational design of Cu_2O-SnO_2 core-shell catalyst for highly selective CO_2-to-CO conversion. *Chemcatchem* **2019**, 11, 4147-4153.

S5. Zeng, J., Bejtka, K., Ju, W., Castellino, M., Chiodoni, A., Sacco, A., Farkhondehfal, M.A., Hernández, S., Rentsch, D., Battaglia, C., and Pirri, C.F. Advanced Cu-Sn foam for selectively converting CO_2 to CO in aqueous solution. *Appl. Catal. B: Environ.* **2018**, 236, 475-482.

S6. Zhao, Y., Wang, C., and Wallace, G.G. Tin nanoparticles decorated copper oxide nanowires for selective electrochemical reduction of aqueous CO_2 to CO. *J. Mater. Chem. A* **2016**, 4, 10710-10718.

S7. Ju, W., Zeng, J., Bejtka, K., Ma, H., Rentsch, D., Castellino, M., Sacco, A., Pirri, C.F., and Battaglia, C. Sn-Decorated Cu for Selective Electrochemical CO_2 to CO Conversion: Precision Architecture beyond Composition Design. *ACS Appl. Energy Mater.* **2019**, 2, 867-872.

S8. Wang, X., Lv, J., Zhang, J., Wang, X.-L., Xue, C., Bian, G., Li, D., Wang, Y., and Wu, T. Hierarchical heterostructure of SnO_2 confined on CuS nanosheets for efficient electrocatalytic CO_2 reduction. *Nanoscale* **2020**, 12, 772-784.

S9. Ju, W., Jiang, F., Ma, H., Pan, Z., Zhao, Y.-B., Pagani, F., Rentsch, D., Wang, J., and Battaglia, C. Electrocatalytic Reduction of Gaseous CO_2 to CO on Sn/Cu-Nanofiber-Based Gas Diffusion Electrodes. *Adv. Energy Mater.* **2019**, 9, 1901514.

S10. Sarfraz, S., Garcia-Esparza, A.T., Jedidi, A., Cavallo, L., and c, K. Cu-Sn Bimetallic Catalyst for Selective Aqueous Electroreduction of CO_2 to CO. *ACS Catal.* **2016**, 6, 2842-2851.

S11. Li, Q., Fu, J., Zhu, W., Chen, Z., Shen, B., Wu, L., Xi, Z., Wang, T., Lu, G., Zhu, J.-j., and Sun, S. Tuning Sn-Catalysis for Electrochemical Reduction of CO_2 to CO via the Core/Shell Cu/SnO_2 Structure. *J. Am. Chem. Soc.* **2017**, 139, 4290-4293.

S12. Morimoto, M., Takatsuji, Y., Yamasaki, R., Hashimoto, H., Nakata, I., Sakakura, T., and Haruyama, T. Electrodeposited Cu-Sn Alloy for Electrochemical CO_2 Reduction to CO/$HCOO^-$. *Electrocatalysis* **2018**, 9, 323-332.

S13. Schreier, M., Héroguel, F., Steier, L., Ahmad, S., Luterbacher, J.S., Mayer, M.T., Luo, J., and Grätzel, M. Solar conversion of CO_2 to CO using Earth-abundant electrocatalysts prepared by atomic layer modification of CuO. *Nature Energy* **2017**, 2, 17087.

S14. Huo, S., Weng, Z., Wu, Z., Zhong, Y., Wu, Y., Fang, J., and Wang, H. Coupled Metal/Oxide Catalysts with Tunable Product Selectivity for Electrocatalytic CO_2 Reduction. *ACS Appl. Mater. Interfaces* **2017**, 9, 28519-28526.

S15. Xiong, W., Yang, J., Shuai, L., Hou, Y., Qiu, M., Li, X., and Leung, M.K.H. CuSn Alloy Nanoparticles on Nitrogen-Doped Graphene for Electrocatalytic CO_2 Reduction. *ChemElectroChem* **2019**, 6, 5951-5957.

S16. Hou, X., Cai, Y., Zhang, D., Li, L., Zhang, X., Zhu, Z., Peng, L., Liu, Y., and Qiao, J. 3D core-shell porous-structured Cu@Sn hybrid electrodes with unprecedented selective CO_2-into-formate electroreduction achieving 100%. *J. Mater. Chem. A* **2019**, 7, 3197-3205.

S17. Wang, P., Qiao, M., Shao, Q., Pi, Y., Zhu, X., Li, Y., and Huang, X. Phase and structure

engineering of copper tin heterostructures for efficient electrochemical carbon dioxide reduction. *Nat. Commun.* **2018**, 9, 4933.

S18. Peng, L., Wang, Y., Masood, I., Zhou, B., Wang, Y., Lin, J., Qiao, J., and Zhang, F.-Y. Self-growing Cu/Sn bimetallic electrocatalysts on nitrogen-doped porous carbon cloth with 3D-hierarchical honeycomb structure for highly active carbon dioxide reduction. *Appl. Catal. B: Environ.* **2020**, 264, 118447.

S19. Ye, K., Cao, A., Shao, J., Wang, G., Si, R., Ta, N., Xiao, J., and Wang, G. Synergy effects on Sn-Cu alloy catalyst for efficient CO_2 electroreduction to formate with high mass activity. *Science Bulletin* **2020**, 65, 711-719.

S20. Wang, J., Zou, J., Hu, X., Ning, S., Wang, X., Kang, X., and Chen, S. Heterostructured intermetallic CuSn catalysts: high performance towards the electrochemical reduction of CO_2 to formate. *J. Mater. Chem. A* **2019**, 7, 27514-27521.

S21. Wang, J., Ji, Y., Shao, Q., Yin, R., Guo, J., Li, Y., and Huang, X. Phase and structure modulating of bimetallic CuSn nanowires boosts electrocatalytic conversion of CO_2. *Nano Energy* **2019**, 59, 138-145.

S22. P. Iyengar, M. J. Kolb, J. R. Pankhurst, F. Calle-Vallejo, R. Buonsanti, *ACS Catal.* **2021**, 11, 4456.

S23 L. R. L. Ting, O. Piqué, S. Y. Lim, M. Tanhaei, F. Calle-Vallejo, B. S. Yeo, *ACS Catal.* **2020**, 10, 4059.

S24 T. T. H. Hoang, S. Verma, S. Ma, T. T. Fister, J. Timoshenko, A. I. Frenkel, P. J. A. Kenis, A. A. Gewirth, *J. Am. Chem. Soc.* **2018**, 140, 5791.

S25 Y. Feng, Z. Li, H. Liu, C. Dong, J. Wang, S. A. Kulinich, X. Du, *Langmuir* **2018**, 34, 13544.
